# Improving Burns and Plastic Surgery Induction Programmes: A Departmental Quality Improvement Project

**DOI:** 10.7759/cureus.51452

**Published:** 2024-01-01

**Authors:** Munir Abukhder, Elliot Ismail, Thomas Dobbs

**Affiliations:** 1 Burns and Plastic Surgery, Morriston Hospital, Swansea, GBR

**Keywords:** education and training, education, burns and plastic surgery, medical-education, continuous medical education

## Abstract

Background: Changes to the undergraduate medical curriculum now offer a greater focus on community-based teaching, communication skills and medical humanities. Unfortunately, this has been at the expense of surgical teaching. The senior house officer is usually the first port of call when a patient is being referred to a plastic surgery department. Therefore, a reasonable level of knowledge is required with regard to emergency presentations, examination skills, and clinical skills to appropriately manage the injury. The primary aims of this quality improvement project are to firstly improve the newly starting doctor’s confidence in undertaking an on-calls in either trauma or burns following the induction programme and to also improve their level of satisfaction.

Methods: The Quality Improvement Project (QIP) team consisted of a Foundation Year 2 doctor, a core surgical trainee, and a registrar. Three Plan, Do, Study, Act (PDSA) cycles were completed to improve the quality of the induction programme. In the first PDSA cycle, junior doctors were provided with a handbook that covered necessary topics regarding burns and plastic surgery. In the second cycle, a structured presentation which included case-based discussions, was incorporated into the trauma aspect of the induction. Finally, in the third cycle, a structured presentation which included case-based discussions, was incorporated into the burns aspect of the induction. Data was collected in the form of a questionnaire one month following the departmental induction for each cycle. The questionnaire assessed the doctor’s confidence levels and degree of satisfaction with the induction programme. Students were also given the opportunity to complete written descriptive feedback at the end of the questionnaire. Furthermore, pre- and post-induction questionnaires on the day of induction for the December and April cohort of doctors were also obtained.

Results: A total of 16 doctors completed the questionnaires. Overall satisfaction, confidence in undertaking trauma on-calls, and confidence in undertaking burns on-calls improved from 3.84/5, 1.83/5, and 2.67/5 in the first cycle to 4.6/5, 3.6/5, and 3.6/5 in the third cycle, respectively. Satisfaction with the clinical emergencies and case discussions aspect of the induction programme improved from 2.17/5 in the first cycle, to 4.6/5 in the third cycle. With regards to the pre- and post-induction questionnaire on the day of induction, the December cohort's correct answer percentage improved from 58.3% to 94.4%, and the April cohort improved from 47.2% to 93.3%.

Conclusion: Whilst it is unlikely to completely prepare new junior doctors for the transition into clinical practice in a unique speciality such as burns and plastic surgery, our study highlights the value of a thorough, multi-stage induction in ensuring junior doctors feel confident to deliver high quality and safe patient care.

## Introduction

Whilst changes to the undergraduate medical curriculum now offer a greater focus on community-based teaching, communication skills and medical humanities, this has been at the expense of surgical teaching [1,2]. Amongst the surgical specialities which have been negatively affected by these reforms is plastic surgery. A survey completed at the University of Birmingham Medical School by a total of 171 students demonstrated that around 70% of medical students chose the media as their main source of knowledge relating to plastic and reconstructive surgery [3]. This lack of knowledge translates into post-graduate skills, with it being shown that newly qualified doctors have deficient knowledge with regard to wound management [4]. These studies highlighted the problem with the lack of inclusion of plastic surgery within the undergraduate medical curriculum.

The senior house officer is usually the first port of call whenever a patient is referred to a plastic surgery department. Therefore, a reasonable level of knowledge is required regarding emergency presentations, examination skills, and clinical skills to appropriately manage the injury. However, as highlighted by previous studies, junior trainees may feel underprepared when they are on-call. The primary aims of this quality improvement project were, therefore, to firstly improve the newly starting doctor’s confidence in undertaking on-calls in either trauma or burns following the induction programme, and secondly to improve the level of satisfaction with the induction programme.

## Materials and methods

The Welsh Centre for Burns and Plastic Surgery, based in Morriston Hospital, offers world-leading care to a population of 10 million. It treats roughly 750 burn patients a year and performs more than 6,500 plastic surgery cases for trauma, cancer, infections or birth defects [5]. The departmental induction programme prior to commencing this quality improvement project (QIP) was an informal face-to-face discussion on the expectations and responsibilities of the role, which included a departmental tour to show juniors the different areas of clinical work, or where they might find equipment essential to the job. Additionally, a confounding factor was apparent that when junior doctors were placed on plastic surgery as their first job within a new hospital or trust; the issues of swipe card access, IT logins and ID badges were also present, making the transitional period more difficult.

The QIP team consisted of a Foundation Year 2 doctor, a core surgical trainee, and three registrars. Since this induction session was targeted at house/senior house officers, the two most junior doctors within the team were able to highlight areas which could potentially be improved through their experience in the role. The senior member of the team ensured that the knowledge within the sessions was correct and accurate.

Plan, Do, Study, Act (PDSA) cycles are widely used for quality improvement projects in most healthcare systems. These cycles provide a structure for iterative testing of changes to improve the quality of the present healthcare systems [6,7]. Our QIP underwent three PDSA cycles in order to improve the quality of the induction programme.

PDSA cycle 1 (August cohort of doctors 2021)

This cohort of junior doctors was provided with a handbook that covered necessary topics, such as hand examination and the management of various plastic surgery-related emergencies. The induction lasted two hours and was without visual aids. Additionally, senior nurses, nurse practitioners and consultants were invited to welcome the new doctors and provide information on their roles and availability for clinical support. They were also provided with a departmental tour. During the first week, the junior doctors were ‘doubled up’ with another member of the team for support.

PDSA cycle 2 (December cohort of doctors 2021)

In addition to the changes made in cycle 1, the induction was separated into ‘burns’ and ‘trauma’. Burns-related teaching remained informal, however, a PowerPoint session titled 'What to expect on a trauma on-call' was delivered covering basic anatomy, examination skills, and management of clinical cases. The session was delivered through case-based discussion, with the help of visual aids to contextualise the teaching.

PDSA cycle 3 (April cohort of doctors 2022)

In addition to the changes made in cycles 1 and 2, additional PowerPoint sessions titled 'What to expect on a burns on-call', and 'What to expect on a wards and plastics dressing clinic shift' were delivered. Similar to the previous cycles, the session was delivered through case-based discussion, with the help of visual aids to contextualise the teaching. The induction length of time increased to three hours.

Data was collected in the form of a questionnaire one month following the departmental induction for each cycle. The questionnaire assessed the doctor’s confidence levels and degree of satisfaction with the induction programme. Students were also given the opportunity to complete written descriptive feedback at the end of the questionnaire. They were asked how to improve the induction for future doctors based on their individual experiences. Furthermore, pre- and post-induction questionnaires on the day of induction for the December and April cohort of doctors were also obtained. 

## Results

A total of six doctors completed the questionnaire for cycle 1, five doctors for cycle 2, and five doctors for cycle 3. Tables 1-2 summarise the grade of the doctors, and if they had any prior experience in plastic surgery.

**Table 1:** Level/grade of the junior doctors

Grade	Cycle 1	Cycle 2	Cycle 3
Core surgical trainee year 2	1	-	-
Core surgical trainee year 1	2	1	2
Foundation year 2	2	4	2
Foundation year 1	1	-	1

**Table 2:** Prior experience in burns and plastic surgery

Prior burns and plastic surgery experience	Cycle 1	Cycle 2	Cycle 3
0 months	5	4	4
1-4 months	1	1	-
>4-8 months		-	-
>8-12 months		-	1

The implementation of a handbook and ‘doubling up’ doctors in cycle 1 was received well by the junior doctors, who reported an overall satisfaction of 3.84/5. However, confidence levels remained low for both trauma and burns on-calls (1.83/5 and 2.67/5, respectively). The subsequent changes made in cycle 2 resulted in an improvement in overall satisfaction with the induction programme from 3.84/5 to 4.6/5 (p-value=0.086). Confidence in being on-call for trauma increased from 1.83/5 to 3.8/5 (p-value <0.001). The implementation of the structured burns teaching within cycle 3 improved confidence in undertaking burn on-calls from 2.67/5 to 3.6/5 (p-value=0.204). Satisfaction in clinical emergency teaching also improved from 2.17/5 to 4.6/5 (p-value < 0.05) between cycles 1 and 3. Table 3 summarises the findings from the questionnaire after each cycle.

**Table 3:** Questionnaire results after each PDSA cycle * Achieved statistical significance (p <0.05) ** 1. Extremely unsatisfied 2. Unsatisfied 3. Neither satisfied or unsatisfied 4. Satisfied 5. Extremely satisfied *** 1. Extremely unconfident 2. Unconfident 3. Neither confident or unconfident 4. Confident 5. Extremely confident PDSA: Plan, Do, Study, Act; SHO: senior house officer, SD: standard deviation; NR: not recorded

Question	PDSA cycle 1 (n=6)			PDSA cycle 2 (n=5)			PDSA cycle 3 (n=5)		
	Mean score (out of 5)	Range	SD	Mean score (out of 5)	Range	SD	Mean score (out of 5)	Range	SD
Overall satisfaction with the induction programme **	3.84	3-5	0.753	4.6	4-5	0.548	4.6	4-5	0.548
How confident are you being the on-call SHO for trauma? ***	1.83	1-3	0.753	3.8*	3-4	0.447	3.6	3-5	0.894
How confident are you being the on-call SHO for burns? ***	2.67	2-3	0.516	NR	NR	NR	3.6	2-5	1.342
How satisfied are you with induction handbook? **	4	3-5	0.632	4.2	3-5	0.837	4.4	4-5	0.548
How satisfied are you with the allocated time? **	4.5	4-5	0.548	4.2	3-5	1.095	4	3-5	0.707
How satisfied are you with the clinical emergencies and scenarios teaching? **	2.17	1-3	0.983	3.8	3-4	0.447	4.6*	3-5	0.894

Written descriptive feedback obtained from the doctors praised how the induction programme consisted of case-based discussions on urgent and non-urgent cases. They did highlight, however, that they were not given a chance to practice clinical skills. They felt that the incorporation of a clinical skills element to the induction programme would further increase the confidence and satisfaction levels amongst the junior doctors. Regarding the pre- and post-induction questionnaire collected on the day of induction, the December cohort's correct answer percentage improved from 58.3% to 94.4% (p-value=<0.05), and the April cohort improved from 47.2% to 93.3% (p-value=<0.05).

## Discussion

As surgical teaching in medical schools has declined over the years, departments have begun to focus on the quality of their induction programmes. However, as demonstrated by previous studies, junior doctors feel underprepared when they are due to commence their burns and plastic surgery rotations. This is likely to have consequences down the line as the on-call senior house officer is expected to discuss referrals and take appropriate action depending on the level of urgency. Therefore, it is crucial that departmental inductions ensure junior doctors are equipped with the necessary information to make correct clinical decisions. Such necessary equipment should include at a basic level the opportunity to become familiar with the geography and logistics of how their local department is run. Clinically, however, junior doctors should also have clear knowledge of basic management, and awareness of their own scope of practice. Senior house officers are more comfortable in their own clinical practice when they are aware of clinical presentations and when is appropriate to escalate to their seniors, but also an awareness of what is expected of them, by way of basic procedural and assessment skills [8]. This is not only paramount to junior doctor confidence, but also to patient safety.

Junior doctors face many hurdles when starting a new role, such as knowledge, of expected responsibilities, department layout, computer system access, provision of identification badges, annual leave policies, and handover instructions. Standard trust inductions may not address all the aforementioned aspects, resulting in unnecessary additional stress when starting a new role [9]. Therefore, it is crucial that the induction programme covers these issues adequately, thus alleviating some of this stress, and easing the transition period. We ensured that our induction programme covered the above information by providing a guided tour of the department and organising introductions to key members of the team such as consultants, advanced nurse practitioners, and the rota co-ordinator. Policies regarding annual leave and sick leave were explained in detail, and relevant contact information was distributed at the end of the session.

Shadowing a colleague with experience in a speciality prior to commencing the job role may be a useful tool which can be utilised to instil confidence in new starting doctors. Previous studies looking into the benefits of shadowing for Foundation Year 1 doctors have demonstrated this to be a highly valuable resource [10,11]. Furthermore, in a study performed by Aryasomayajula et al., junior doctors who received appropriate teaching in theory and practical skills, along with shadowing experience, went to later on be able to run their own acute ENT clinics [12]. We adopted a similar approach where new doctors were paired with a senior house officer who already had some experience within the role. This was received well by the doctors in each rotation, stating that it allowed them to reflect on their colleagues' approach. This ‘doubling up’ experienced doctor was based on educational theory, namely Vygotsky’s theory of cognitive development [13]. The senior house officer whom our new inductees pair up with takes the role of a ‘more knowledgeable other’, instigating collaborative learning and moving them into their 'Zone of Proximal Development' which facilitates rapid growth in the new doctors’ confidence and knowledge. It is worth noting however that the shadowing experience may differ from doctor to doctor. This modality is dependent on the willingness of the senior house officer to engage with the new doctor, finding useful activities for them to undertake, and providing them with useful feedback. However, feedback can at times be irrelevant or unstructured. Although we did not receive negative feedback regarding this intervention, we did not specifically ask for it. Training senior house officers to provide structured feedback with specific learning objectives in mind would likely improve the experience of the new doctors, as the feedback will likely match the needs of the trainee.

Thomson et al. recommended that departmental inductions for Foundation Year 1 doctors should follow a standardised format, where the content of the induction is monitored; they also recommended involving Foundation Year 1 doctors in determining the content of the induction [14]. We adhered to these recommendations throughout all three QIP cycles by ensuring our interventions were guided by the feedback received from the outgoing junior doctors. Outgoing doctors are likely to be the best to know what they would need in order to optimally perform in their expected role; therefore, their advice would be the most relevant to the new doctors. The senior members of the team reviewed the content prior to delivering the programme, therefore ensuring that the content was of good quality.

It is established that a key factor in safe patient care is appropriate and timely escalation to seniors, and that failure to do so will have detrimental effects on patient safety [15]. Rotella et al. discuss factors found to contribute to junior doctors’ confidence in raising clinical concerns. One of the largest aspects is junior doctors’ familiarity with clinical scenarios and presentations, and knowledge of management plans [8]. We have evidenced this further; the introduction of case-based discussion in induction sessions, and the usage of a clinical handbook outlining key presentations and management increased confidence to face an on-call shift by almost 100% (1.83/5 to 3.6/5). Confident junior doctors will ultimately increase patient safety and outcomes.

This quality improvement project is not without its limitations. Firstly, our sample size is very small, therefore reducing the validity, and as a consequence, the generalisability of our results. The absence of pre-audit data pertaining to confidence and satisfaction levels is a further limitation in our study. Although there was an overall increase in confidence levels following each PDSA cycle, confidence levels for being on-call for both trauma and burns were particularly low following the first PDSA cycle. This finding may have been specific to the first cohort of doctors only, or it may have been a reflection of the quality of our teaching programme. Pre-audit data would have assisted in interpreting these results. An additional limitation within our study lies in the absence of data regarding the educational institutions attended by our cohort of doctors, as well as whether their undergraduate curriculum incorporated dedicated burns and plastic surgery teaching or not. This information would have likely provided further insight into the potential variability in undergraduate surgical teaching and the consequences it may have on the confidence of junior doctors undertaking their first burns and plastic surgery rotation. Finally, the absence of a control group within our study weakens our ability to attribute our findings to the interventions implemented. 

## Conclusions

Whilst it is unlikely to completely prepare new junior doctors for the transition into clinical practice in a unique speciality such as burns and plastic surgery, our study displays the value of a thorough, multi-stage induction to ensure that junior doctors are confident in their abilities to deliver safe and high-quality patient care. The initial findings of this research indicated that departmental induction was lacking and not currently meeting junior doctor’s needs. However, we have shown through three PDSA cycles that through using tools such as handbooks, ‘doubling up’ with more experienced doctors, and case-based discussions specific to the speciality, departments can create well-educated and supported junior doctors.
